# AutoML accurately predicts endovascular mechanical thrombectomy in acute large vessel ischemic stroke

**DOI:** 10.3389/fneur.2023.1259958

**Published:** 2023-09-28

**Authors:** Rishi Raj, Santhosh Kumar Kannath, Jimson Mathew, P. N. Sylaja

**Affiliations:** ^1^Department of Computer Science and Engineering, Indian Institute of Technology Patna, Patna, India; ^2^Department of Imaging Sciences and Interventional Radiology, Sree Chitra Tirunal Institute for Medical Sciences and Technology, Trivandrum, Kerala, India

**Keywords:** autoML, traditional ML, large vessel occlusion, mechanical thrombectomy, mRS score

## Abstract

**Background and objective:**

Automated machine learning or autoML has been widely deployed in various industries. However, their adoption in healthcare, especially in clinical settings is constrained due to a lack of clear understanding and explainability. The aim of this study is to utilize autoML for the prediction of functional outcomes in patients who underwent mechanical thrombectomy and compare it with traditional ML models with a focus on the explainability of the trained models.

**Methods:**

A total of 156 patients of acute ischemic stroke with Large Vessel Occlusion (LVO) who underwent mechanical thrombectomy within 24 h of stroke onset were included in the study. A total of 34 treatment variables including clinical, demographic, imaging, and procedure-related data were extracted. Various conventional machine learning models such as decision tree classifier, logistic regression, random forest, kNN, and SVM as well as various autoML models such as AutoGluon, MLJAR, Auto-Sklearn, TPOT, and H2O were used to predict the modified Rankin score (mRS) at the time of patient discharge and 3 months follow-up. The sensitivity, specificity, accuracy, and AUC for traditional ML and autoML models were compared.

**Results:**

The autoML models outperformed the traditional ML models. For the prediction of mRS at discharge, the highest testing accuracy obtained by traditional ML models for the decision tree classifier was 74.11%, whereas for autoML which was obtained through AutoGluon, it showed an accuracy of 88.23%. Similarly, for mRS at 3 months, the highest testing accuracy of traditional ML was that of the SVM classifier at 76.5%, whereas that of autoML was 85.18% obtained through MLJAR. The 24-h ASPECTS score was the most important predictor for mRS at discharge whereas for prediction of mRS at 3 months, the most important factor was mRS at discharge.

**Conclusion:**

Automated machine learning models based on multiple treatment variables can predict the functional outcome in patients more accurately than traditional ML models. The ease of clinical coding and deployment can assist clinicians in the critical decision-making process. We have developed a demo application which can be accessed at https://mrs-score-calculator.onrender.com/.

## 1. Introduction

Deployment of machine learning (ML) in the healthcare domain has helped in improving outcomes, cutting costs, and advancing clinical research and understanding (1). However, their wide adoption is constrained by data accessibility, dataset imbalances, requirement of data science expertise in fine-tuning and model deployment (2). AutoML is an attempt to solve the above issues, not constrained by domain, to minimize human intervention in data preprocessing, feature selection, and model development and deployment. While traditional artificial intelligence (AI) requires expertise in model development and deployment, autoML aims to make the technology more decentralized and accessible (3). AutoML is a serious attempt at algorithmic automation with support for explainability so that cross-domain experts without specialized knowledge of AI can benefit from the advancements. In this study, we aim to investigate the performance of autoML vis-a-vis traditional ML approaches with a focus on understanding internal processes and ease of clinical coding to apprise the clinical community. Specifically, we aim to predict the clinical outcome of stroke thrombectomy, which would help clinicians in better decision-making for the invasive high-risk procedure. The above application study has been selected as it represents the general complexity and challenges in the medical domain, such as a small sample size, a high dataset imbalance, and a large number of feature variables. To the best of our knowledge, there are no previous studies investigating the performance of autoML in clinical studies.

Ischemic stroke due to occlusion of a major intracranial vessel, also called “large vessel occlusion” (LVO) is the most common cause of neurological impairment worldwide (4). The occlusion is due to the formation of a thrombus within one or more of the major intracranial vessels (5). After coronary artery disease, stroke ranks as the second leading cause of death. It is the third leading cause of early mortality, causing the death of an estimated 6.2 million people annually and 113 million disability-adjusted life years (DALY) (6). Although, a well-timed medical management of stroke including intravenous thrombolysis therapy for dissolution of the intravascular thrombus is the mainstay of treatment, a subset of stroke patients also benefit from endovascular treatment in which the occluding thrombus is directly removed via trans-arterial approach, thereby recanalizing the vessel and saving the brain tissues from irreversible damage. This procedure called mechanical thrombectomy is invasive and causes various complications (7). The factors predicting the functional outcome of the thrombectomy procedure are unclear. While many patients benefit from the procedure, few show neurological deterioration despite undergoing the mechanical thrombectomy procedure (4). Therefore, it would be ideal to understand the factors influencing the functional outcome post-mechanical thrombectomy. However, this is challenging as a wide range of factors influence the post-procedure outcome.

**Figure 1:**
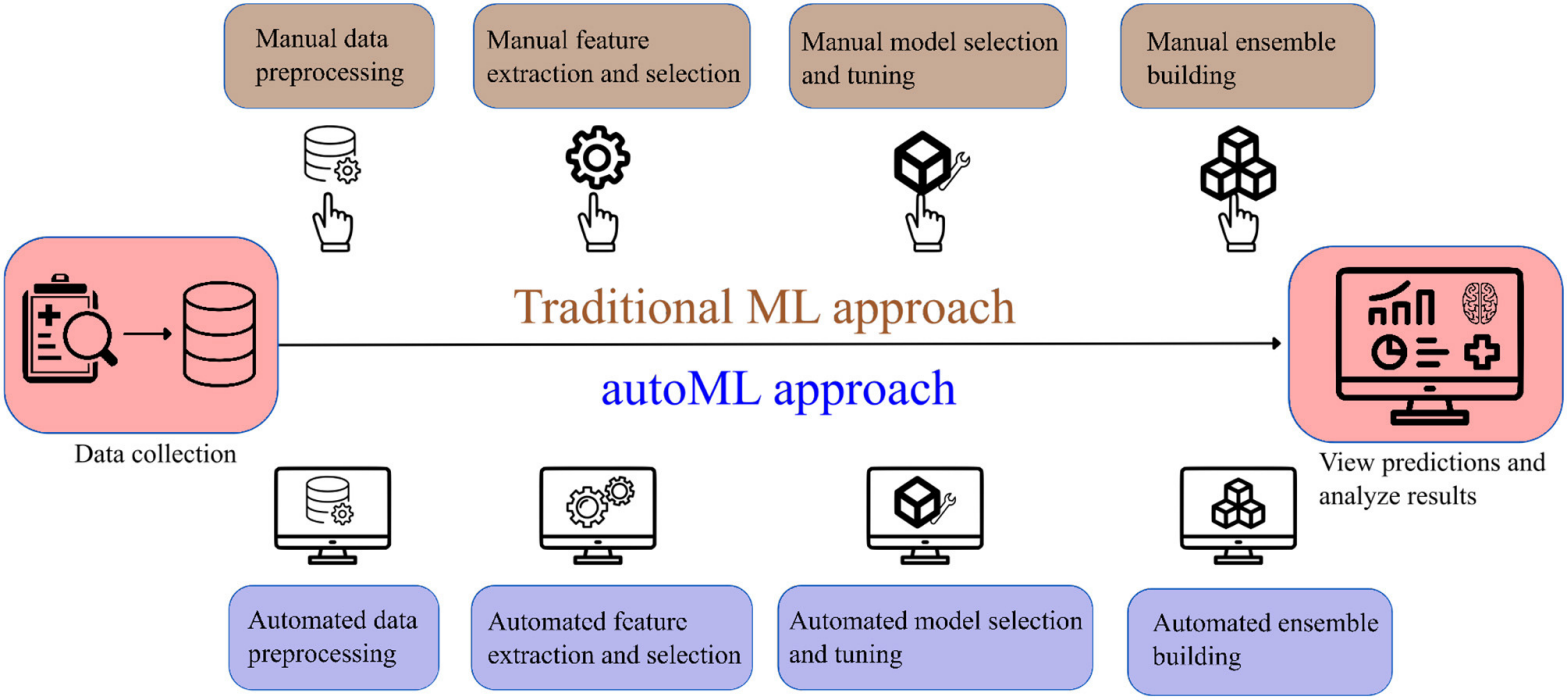
Simplified flowchart describing the difference in traditional ML and autoML approaches.

Few studies have been undertaken to predict post-mechanical thrombectomy functional outcomes in the patients of LVO. Heo et al. (8) developed three machine learning models (neural network, random forest, and logistic regression) to predict the modified Rankin Scale (mRS) score at 3 months. Similarly, Brugnara et al. (9) compared different machine learning models with conventional statistical methods to predict the mRS score at 3 months. Although these models have achieved good accuracy, they have their own limitations such as manual feature selection, extraction, and lack of explainability. We investigate if autoML can be an alternative solution to the limitations of traditional ML models. AutoML refers to the class of algorithms that focus on the gradual automation of machine learning by rule-based improvements in the design and optimization of various machine learning algorithms (10). AutoML is a scalable, efficient, and easily deployable solution that automates time-consuming iterative tasks of model selection and development. It also constructs an ensemble of ML algorithms and constructs the best-performing ML method with given data (11). Figure 1 shows a simplified flowchart describing the difference between traditional ML and autoML approaches.

The primary contributions of this study are:

- Investigating the prediction capability and explainability of autoML algorithms vis-a-vis traditional ML frameworks in the clinical domain.
- Assessing the application of autoML models as a potential clinical decision support tool for predicting functional outcomes in patients who underwent mechanical thrombectomy.
- Considering an exhaustive list of 34 treatment variables for the AI-based prediction from a clinical perspective.

## 2. Materials and methods

The study was approved by the Ethical Committee of Sree Chitra Tirunal Institute of Medical Sciences and Technology, Trivandrum, Kerala, India. As it was a retrospective study, the need for informed consent was waived.

### 2.1. Definition of the predicted outcome

The modified Rankin score (mRS score) at the time of the patient's discharge from the hospital and 3 months follow-up was used to determine the functional outcome of mechanical thrombectomy. The mRS score is a 6-point disability score with possible scores from zero to six where zero denotes lack of any symptoms, five denotes severe bedridden disability and a score of six denotes death. We classified the mRS score into three classes: class 1 (mRS ≦ 2 = favorable outcome), class 2 (mRS > 2 and ≦ 5 = unfavorable outcome), and class 3 (mRS = 6 = death) (12).

### 2.2. Study cohort

**Figure 2:**
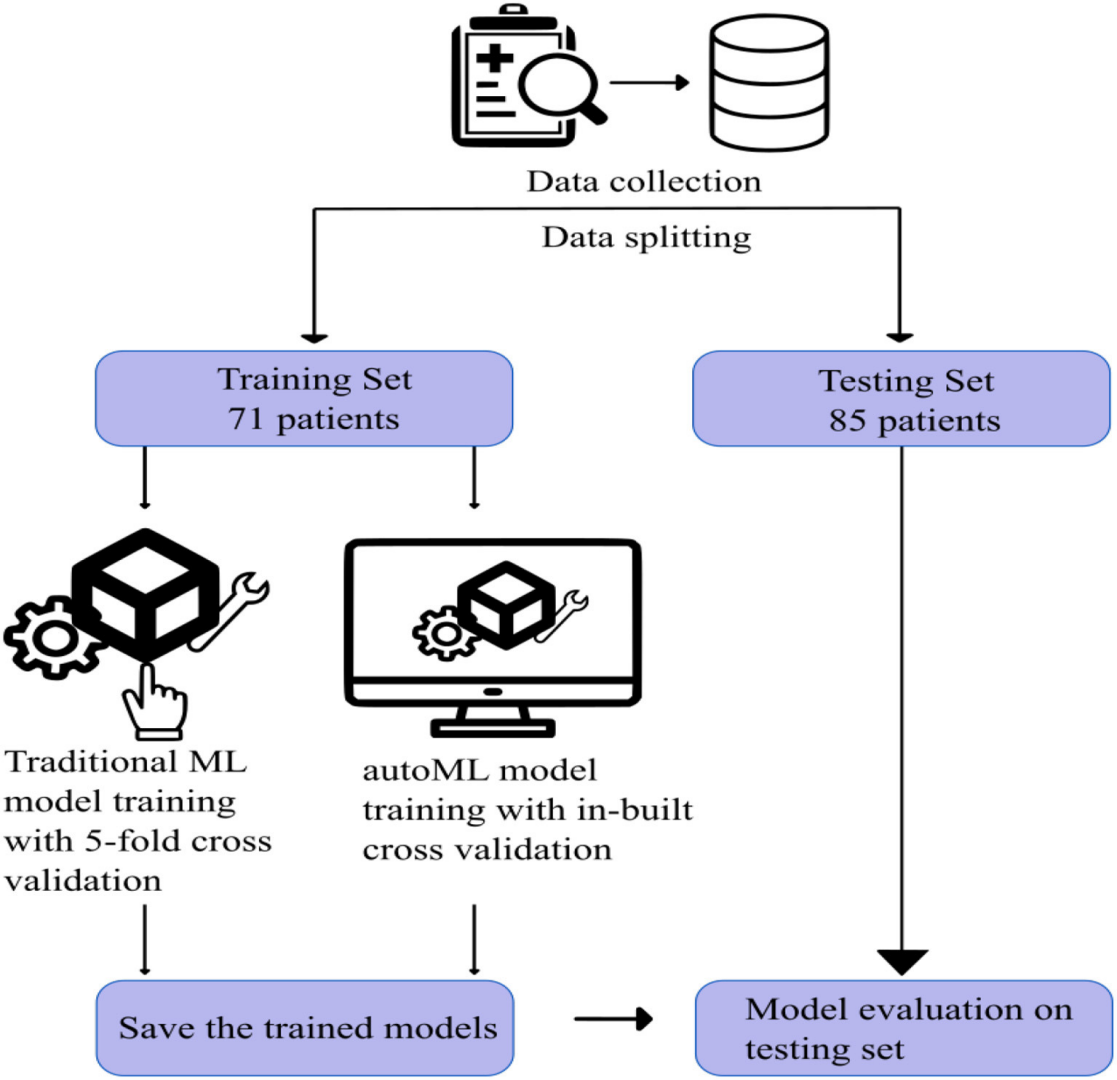
Visual representation of the workflow of the study.

Patient data between January 2013 and January 2022 were retrieved from the institute database. Patients with acute ischemic stroke due to LVO and who subsequently underwent CT evaluation followed by mechanical thrombectomy were included in the study. Patients with posterior circulation stroke, who had no clinical data, and those who did not undergo pretreatment CT imaging were excluded from the study. After applying the inclusion and exclusion criteria, 156 patients were selected for the study. Figure 2 shows the visual representation of the workflow of the study.

For both traditional and autoML models, the remaining 156 patients were grouped randomly into either a training or testing cohort in an approximately 1:1 ratio, which was matched for various prognostic classes (Table 1). The training and testing set had 71 and 85 patients, respectively. The division of the dataset into an approximately 1:1 ratio is a slight departure from the conventional approach of dividing the dataset in the ratio of 7:3. This is done to train the model on a small sample space, replicating the situation in a single-center cohort with existing low data sharing protocol in the medical domain. This also helped us to create a large external dataset of 85 patients to capture the generalizability capacity of the developed model. Dividing the dataset in the ratio of 7:3 would have left us with a small test sample size, thereby not reflecting the diversity of cases in a real-world scenario.

**Table 1:** Selected demographic, clinical, and treatment characteristics and clinical outcome in training and testing set cohorts.

	**Training set**	**Testing set**
Age, mean (range)	60.91 (39–88)	57.35 (23–78)
Females, *n* (%)	28 (39.4)	33 (38.8)
Reached within routine working hours, *n* (%)	36 (50.7)	47 (55.29)
Hypertension, *n*(%)	40 (56.34)	51 (60)
Diabetes, *n* (%)	26 (36.62)	35 (41.17)
Smoking, *n* (%)	16 (22.53)	10 (11.76)
Coronary artery disease, *n* (%)	15 (21.1)	16 (18.8)
Valvular heart disease, *n* (%)	11 (15.4)	21 (24.7)
Dyslipidemia, *n* (%)	15 (21.1)	22 (25.88)
Past Stroke, *n* (%)	9 (12.6)	16 (18.8)
Antiplatelet medications, *n* (%)	18 (25.35)	25 (29.41)
Right Hemisphere, *n* (%)	35 (49.29)	38 (44.70)
NIHSS at admission, median (IQR)	16 (12–20)	16 (11–21)
mRS at admission, median (IQR)	4 (4–4)	4 (4–5)
Baseline CT ASPECTS, median (IQR)	7 (6–8)	6 (5–7)
Hyperdense middle cerebral artery sign, *n* (%)	37 (52.11)	43 (50.58)
**Occluded vessel**		
ICA and beyond, *n* (%)	12 (16.90)	23 (27.05)
M1, MCA and beyond, *n* (%)	58 (81.69)	59 (69.41)
Involvement of anterior cerebral artery, *n* (%)	9 (12.6)	13 (15.29)
Clot burden score, median (IQR)	6 (5–6)	6 (4–6)
Thrombus length, median (IQR)	10 (14–7)	11 (14–8)
Modified tan score, *n* (%)	38 (53.52)	44 (51.76)
Bridging thrombolysis, *n* (%)	27 (38.02)	26 (30.58)
**TICI score**		
0, *n* (%)	1 (1.40)	7 (8.23)
1, *n* (%)	3 (4.22)	4 (4.7)
2a, *n* (%)	7 (9.85)	13 (15.29)
2b, *n* (%)	24 (33.8)	28 (32.94)
2c+3, *n* (%)	36 (50.7)	32 (37.64)
Symptomatic intracerebral bleed, *n* (%)	4 (5.63)	6 (7.05)
24 hour CT ASPECTS score, median (IQR)	6 (5–7)	6 (4–7)
mRS at Discharge, median (IQR)	3 (2-4)	4 (2-4)
mRS at 3 months, median (IQR)	2 (1-3)	3 (1-4)
Time from stroke onset	108	110
to recanalisation, median (range in minutes)	(45–250)	(30–345)

### 2.3. Pretreatment variables

As a wide range of factors are known to interplay in predicting the outcome of thrombectomy procedures, a total of 34 variables including clinical, demography, imaging, and parameters pertaining to intervention procedures were included in our study. The clinical variables included age; gender; risk factors such as diabetes, hypertension, hyperlipidemia, peripheral vascular diseases, smoking, addiction, or cardiac disease; pre-stroke mRS score; blood pressure and blood sugar at presentation; antithrombotic/antiplatelet medication status; NIHSS score at presentation; NIHSS stroke at 24 h post-procedure; stroke etiology; type of stroke; and bridging thrombolysis. The imaging variables comprised ASPECTS score at presentation, presence of hyperdense clot, thrombus length, site of occlusion, CT collateral score (modified TAN score), follow-up ASPECTS score, and hemorrhagic transformation. The variables pertaining to the thrombectomy procedure included the final recanalization score (thrombolysis in cerebral ischemia-TICI), major life-threatening periprocedural events during mechanical thrombectomy, and procedures during or outside routine working hours (8 a.m. to 4 p.m.). Time metrics such as door-to-groin puncture and groin puncture to recanalization were summed into a single value for analysis. To the best of our knowledge, this is the first study encompassing such an exhaustive list of treatment variables for the prediction of a patient's prognosis. All the variables were extracted from the hospital database and verified by the interventional neuroradiologist. Table 1 describes basic statistics of the training and testing datasets.

### 2.4. Data preprocessing

Automated data preprocessing is a crucial aspect of autoML. Various autoMLs employ customized preprocessing techniques that define their prediction capability. For instance, autoGluon (13) identifies the given problem as a classification or regression problem on the basis of the label column. It categorizes each feature as categorical, numeric, text, or date/time and handles them separately. Also, missing values are handled by categorizing them in the “Unknown” category rather than imputing them, which allows autoGluon to handle missing values even during test time. Similar strategies are employed by different autoMLs, which reduce human intervention and save manual effort.

However, we did simple preprocessing to adhere to the clinical literature. The categorical variables were dichotomized for their presence or absence. The ordinal data which included the various scoring systems were grouped under fewer subcategories. For instance,

- ASPECTS scores were grouped into three subcategories as < 5, 5–7, and >7 (14)
- NIHSS scores were < 5, 5–15, and >15 (15)
- clot lengths were ≤ 10 mm, 10–20 mm, and ≥20 mm (16)
- CT collateral score was dichotomized as per modified TAN score (17, 18)
- thrombolysis in cerebral ischemia scores were TICI 0 or 1, TICI 2a or 2b, and TICI 2c or 3 (19)
- The level or site of vessel occlusion was grouped under three classes based on the involvement of the Internal Carotid Artery (ICA), proximal segment (M1), distal segment (M2) of Middle Cerebral Artery (MCA), and Anterior Cerebral Artery (ACA) (20)
  - Class 1: All ICA occlusion
  - Class 2: M1 occlusion without ICA occlusion, with or without M2, and beyond occlusion
  - Class 3: M2 and beyond occlusion without ICA and M1 occlusion.

Additionally, the presence or absence of ACA involvement was also considered due to its role in the collateralization of MCA branches.

### 2.5. Model building

To extensively compare traditional ML models with autoML, we have selected five models for each category. Although the detailed workflow has been explained in respective publications, for the purpose of completeness and understanding, we summarize their workings in a simple manner.

For derivation of the model, the training data could be defined as *X* = {x^(1)^, x^(2)^, x^(3)^, …, x^(n)^}, where *n* is the number of patients in the training data. The individual input vector *x* is defined as *x* = [x_1_, x_2_, x_3_, …, x_m_], where x_1_, …, x_m_ are components of the input vector and *m* is the number of variables included in this study. The target is determined by *Y*.


Y={“favorable outcome,” “unfavorable outcome,” and “death”}

#### 2.5.1. ML frameworks

**Support vector machine** (21): Support Vector Machine or Support Vector Classifier constructs a hyperplane(s) to separate two or more classes. The objective is to construct a hyperplane and position it such that it is at a maximum distance from the data points. An important hyper-parameter is the selection of kernel function. A kernel function reduces the computational burden by efficiently enabling computations to be performed in a higher-dimensional feature space. A few examples of kernel functions are linear function, polynomial function, radial basis function, and sigmoid function. We have selected a radial basis function as our kernel to handle the non-linear data in our sample (22).

**Logistic regression** (23): A logistic regression model is based on a sigmoid function, which outputs a value in the range between zero and one. Let *p* be the probability that the event we want to predict has occurred. Logistic regression assumes that the odds ratio *z* of the event is well explained by the linear function of the adequately weighted variables. *w*_i_ represents the weight of the *i* th variable.


$$\begin{array}{c} z=\log\frac{p}{1-p}=\overset{n}{\underset{i=0}{\sum }}w_{i}x_{i}\\ p=\frac{1}{1+e^{-z}} \end{array}$$

Next, the cost function *J*(*w*) is defined as written below:


(1) $$\begin{array}{l} J(w)=\sum_{i=1}^{n}[-y^{\left(i\right)}\log\left(\phi \left(z^{\left(i\right)}\right)\right)-\\ \;\left(1-y^{\left(i\right)}\right)\log\left(1-\phi \left(z^{\left(i\right)}\right)\right]+\frac{\lambda }{2}R \end{array}$$

The first section of *J*(*w*) was derived from the log-likelihood function of the event *Y*, and the second section, *R*, was the regularization parameter. The hyper-parameters are the regularization parameters, which could be L1 or L2 regularization, and λ, which needs to be set up by hyperparameter tuning. For our experiments, we had set L2 as our regularization function with LBFGS (Broyden-Fletcher-Goldfarb-Shanno) as our solver (24). The number of maximum iterations was set to 1,000.

**k nearest neighbors** (25): kNN, a supervised learning algorithm, uses proximity to determine the class of the test data. It assumes that similar data points exist in close proximity. In this algorithm, k is the hyper-parameter, which decides the number of the closest points that would be considered for predictions. If k is too low, it would lead to overfitting, and if it is too high, it would lead to underfitting. kNN is also sensitive to outliers and is not memory-efficient. For our experiments, we selected the value of k as five.

**Decision tree classifier** (26): A decision tree classifier, uses a set of rules to make decisions that are based on Information Gain (IG). IG is the basic criteria to determine if a feature should be used to split a node for expanding the tree. The information gain (*IG*) in a decision tree is defined as (26):


(2) $$\begin{array}{l} IG\left(D_{p},f\right)=I\left(D_{p}\right)-\frac{N_{\text{left}}}{N_{p}}I\left(D_{\text{left}}\right)-\frac{N_{\text{left}}}{N_{p}}I\left(D_{\text{right}}\right) \end{array}$$

Here, *D*_p_ is the data of the parent node, *D*_left_ is the data of the left child node, and *D*_right_ is the data of the right child node. *I* is the impurity of the data, which is defined either as an entropy or Gini impurity. *N*_p_ is the number of the parent node, *N*_left_ is the number of the left child node, and *N*_right_ is the number of the right child node. When *I* is defined as the “entropy,” *I*_H_ is defined as written below (26):


(3) $$\begin{array}{l} I_{H}\left(t\right)=-\overset{c}{\underset{i=1}{\sum }}p\left(i|t\right)\log_{2}p\left(i|t\right) \end{array}$$

When *I* is defined as the “Gini impurity,” *I*_G_ is defined as stated below (26):


(4) $$\begin{array}{l} I_{G}\left(t\right)=\overset{c}{\underset{i=1}{\sum }}p\left(i|t\right)\left(1-p\left(i|t\right)\right) \end{array}$$

The depth of the tree is determined on the basis of the training dataset. In our experiments, we have selected Gini Impurity as our impurity criterion.

**Random forest classifier** (27): Since decision tree classifiers are prone to overfitting, the creation of ensembles is one of the most popular methods. The algorithm of a random forest classifier can be summarized as:

1. For *t* = 1 to *T* :
  A) Draw a bootstrap sample |*D*| from the dataset *D* and make the bootstrap sample *D*_*t*_.
  B) Select *q* variables at random from the *m* variables.
  C) Grow a random-forest tree *M*_t_ with *D*_t_ until the minimum node size *n*_min_ is reached.
2. Output the ensemble of trees ${\left\{M_{t}\right\}}_{1}^{T}$.
3. For the classification, majority voting is used. When Ŷ_*t*_(*x*) is the class prediction of the *t* th tree of the forest, the prediction is shown below:

(5) $${\hat{Y}}_{rf}^{T}(x)=\text{ majority vote }{\left\{{\hat{Y}}_{t}(x)\right\}}_{1}^{T}\text{. }$$

For our experiments, the number of trees in the forest was set to 100, owing to the large number of features in the dataset. The impurity criterion was the same as the Decision Tree Classifier, that is, the Gini impurity.

#### 2.5.2. autoML frameworks

**Auto-sklearn** (28): Auto-sklearn is one of the earliest AutoML frameworks that aims to solve the Combined Algorithm Selection and Hyperparameter optimization (CASH) problem by introducing two innovations.

Firstly, it introduces a meta-learning step to warm-start the Bayesian Optimization technique. Bayesian optimization fits a probabilistic model to capture the relationship between hyperparameter settings and their measured performance; it then uses this model to select the most promising hyperparameter setting. However, Bayesian optimization is slow for hyperparameter spaces as large as those of entire ML frameworks but can fine-tune performance over time. The introduction of the meta-learning step helps to quickly suggest some instantiations of the ML framework that are likely to perform exceptionally well. Still, it is unable to provide fine-grained information on performance. Integrating the benefits of both techniques makes meta-learning complementary to Bayesian optimization.

**Figure 3:**
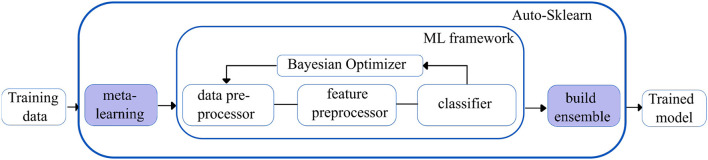
Simplified workflow in the auto-sklearn framework. The boxes in blue indicate the innovations introduced by Auto-sklearn.

Secondly, Auto-sklearn introduces the ensembling of models trained by meta-learning and Bayesian optimization. This ensemble architecture is more resilient and less prone to overfitting as it does not have to adhere to a single pipeline layout. Given the well-known tendency for ensembles to outperform individual models, it can also increase performance. Figure 3 shows an overview of the Auto-Sklearn workflow.

**H2O autoML** (29): The H2O framework (H2O.ai, 2013) contains the automated machine learning algorithm H2O AutoML (H2O.ai, 2017), which handles binary, multi-class classification, and regression tasks on tabular datasets. It supports a variety of basic models, including Deep Neural Networks, Random Forests, Gradient Boosting Machines (GBM), and Generalized Linear Models (GLM). H2O AutoML introduces two essential advancements to improve the models' accuracies. First, the base models are fine-tuned using the fast random search approach (defined as lambda search), in which the hyperparameters are selected from the range of values (defined as alpha values) speculated to be the most crucial. The quick random search method introduced in H2O AutoML is comparable to the auto-sklearn Bayesian optimization method.

**Figure 4:**
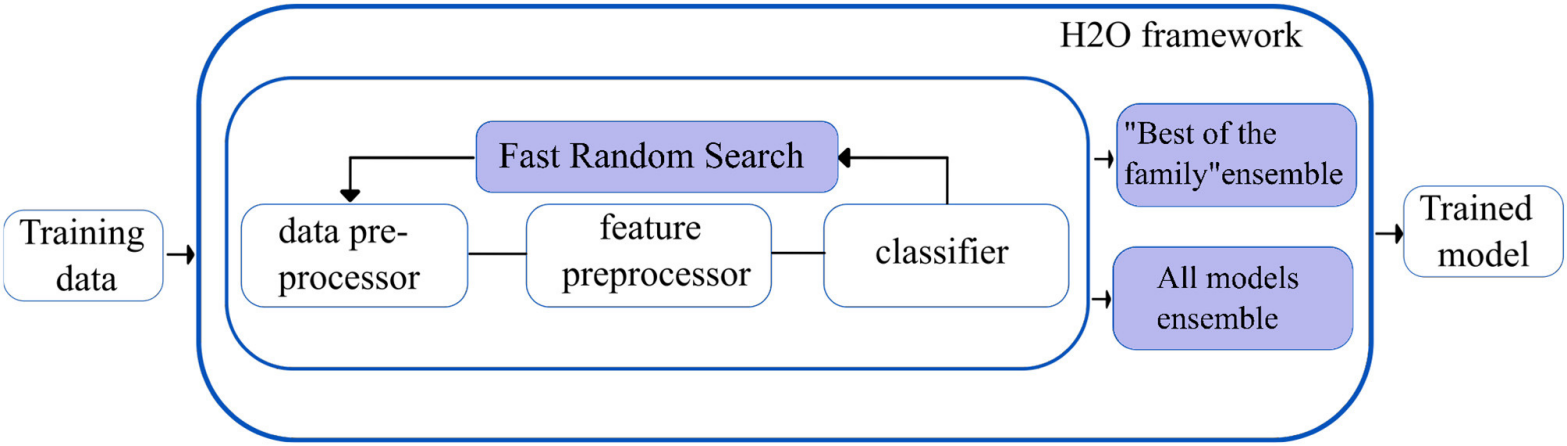
Simplified workflow of the H2O autoML framework. The boxes in blue indicate the innovations introduced by the H2O AutoML framework.

Second, H2O AutoML designs and deploys two stacked models: “All models ensemble,” which combines all the base models trained, and “Best of the Family ensemble,” which contains the best-performing models. Fast random searches across several algorithm families provide a wide range of base models, but when combined with stacking, they yield efficacious ensembles. However, the stacked ensembles perform exceptionally well when the base models are robust separately and have uncorrelated errors. Figure 4 shows the overview of the working of the H2O autoML framework.

**Figure 5:**
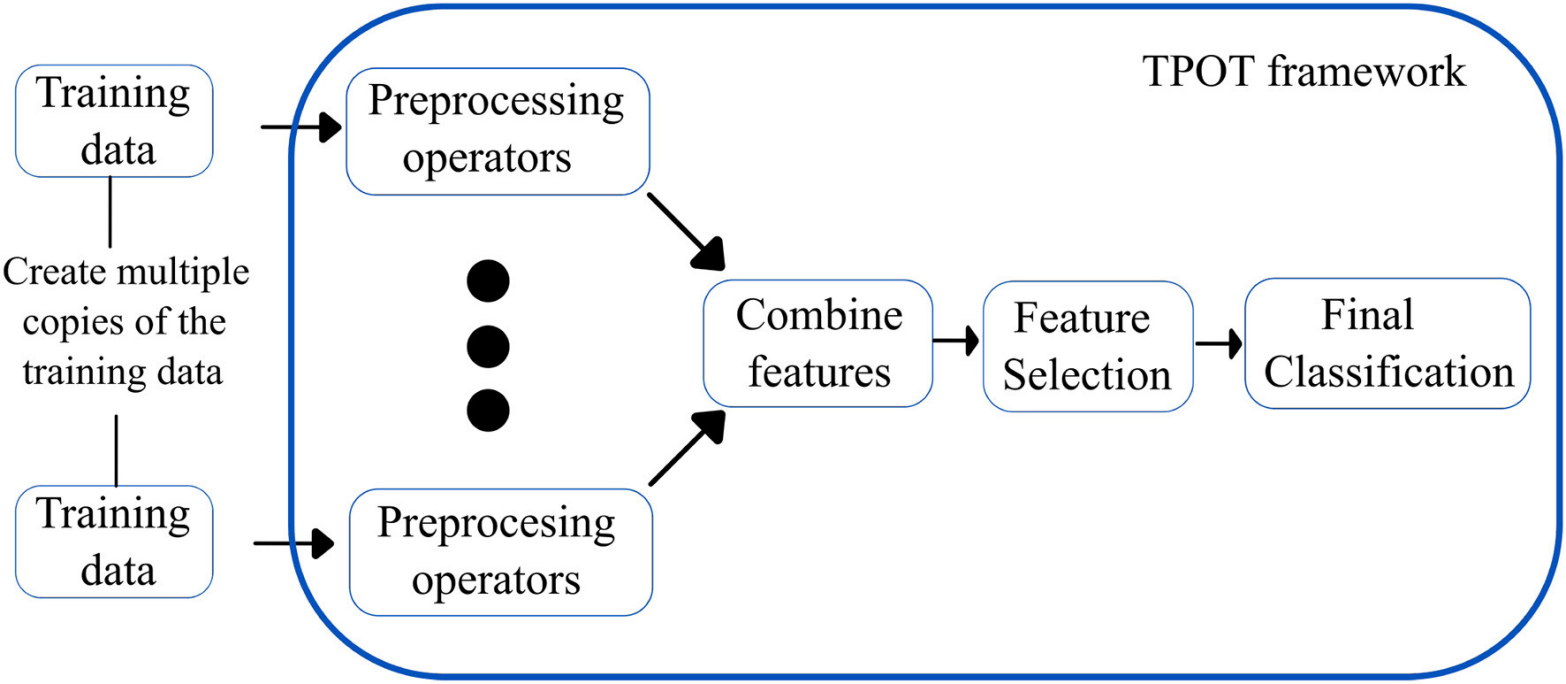
Simplified workflow of the TPOT autoML framework. The boxes in blue indicate the innovations introduced by the TPOT autoML framework.

**Tree-based pipeline optimization tool** (30): TPOT is another popular AutoML based on genetic programming (GP), an evolutionary computation technique for automatically constructing computer programs. It is a wrapper for scikit-learn, the Python machine learning toolkit, that manages feature preprocessing, model selection, and hyperparameter optimization operations for a specific machine learning task. Specifically, TPOT creates multiple copies of the dataset and modifies them using pipeline operators. The pipeline operators have supervised classification operators (e.g., Decision trees, kNN, Random Forests, XGBoost, and Logistic Regression) and feature preprocessing operators (e.g., Scalers, PCA, Binarizers, and Polynomial Features), which create new modified features. The features are then combined to create genetic programming primitives, which are subsequently used to create genetic programming trees. The feature selection operators include Variance Threshold, Select K Best, and Recursive Feature Elimination. The top 20 pipelines with the highest degree of classification accuracy are determined, and five copies of each are rendered to generate a new population. The new population is then subjected to one-point crossover and mutation. The evaluation is performed for 100 generations, adding and adjusting pipeline operators that increase classification accuracy and pruning operators that reduce classification accuracy. Eventually, the algorithm selects the exemplary “best” pipeline from the iteration that has the maximum accuracy. Figure 5 shows the overview of the working of TPOT autoML.

**MLJAR** (31): MLJAR is an AutoML python package that works on tabular data and provides four modes of operation: “explain,” “compete,” “perform,” and “optuna.” While the “Explain” mode is suitable for the dataset's initial data analysis and explainability, the “compete” mode is for model training in time-constraint circumstances. In the “perform” mode, production-ready ML pipelines are deployed based on the optimum prediction time. MLJAR also deploys ML algorithms with hyperparameters optimized as proposed in the “Optuna framework.” The important characteristics of MLJAR are listed below:

1. It establishes a “baseline” for the data so that ML model performance may be accessed. For classification tasks, the “baseline” is calculated using the prior class distribution; for regression tasks, it is calculated using the simple mean.
2. Models are trained using a variety of algorithms, including Nearest Neighbors, Linear, Random Forest, Extra Trees, LightGBM, Xgboost, and CatBoost.
3. Features preprocessing, such as missing values imputation and converting to categoricals, are accomplished inherently.
4. It performs hyperparameters optimization by random search over a defined set of values, optuna framework, and hill-climbing to fine-tune final models.
5. It can design, test, and deploy ensemble models.
6. The most crucial characteristic of MLJAR is its explainability: All ML pipelines created can be analyzed using markdown reports containing details of all models.
7. Feature importance for each feature is computed using permutation, and for every algorithm, dependence and decision plots can be viewed and analyzed.

**AutoGluon** (13): Autogluon is an open source AutoML by awslabs and supports basic algorithms such as LightGBM, LightGBMXT, CatBoost, XGBoost, Random forests, Extremely Randomized Trees, k-Nearest Neighbors, and neural networks. Apart from the basic algorithms, AutoGluon introduces two crucial innovations, which boost the efficiency of the models trained.

Firstly, appropriately tuned neural networks are integrated with the ensembled models, which provide enhanced accuracy gains. Since the decision boundaries learned by neural networks differ from the tree-based models, they introduce variability and generalization to the trained models.

**Figure 6:**
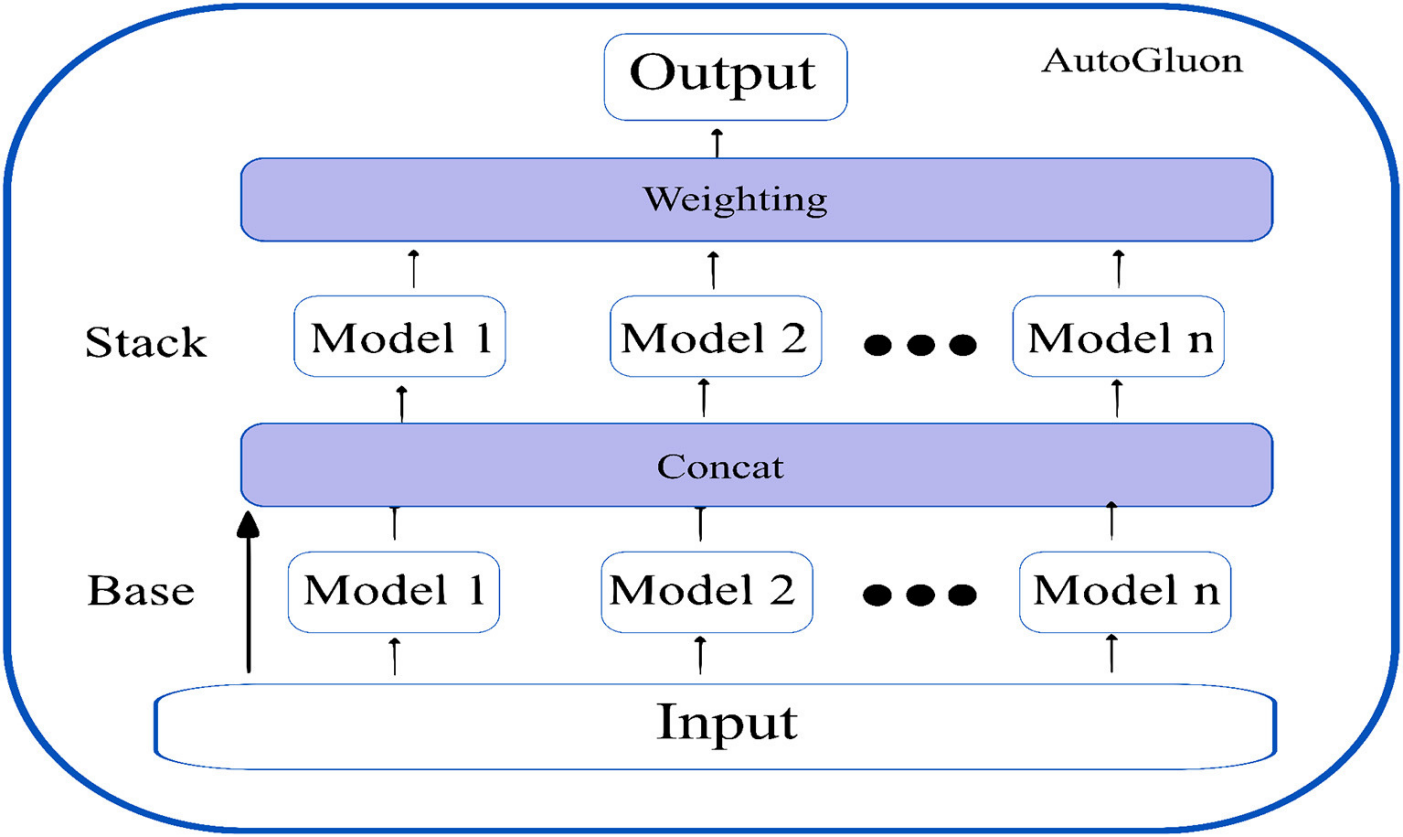
Multi-layer stack ensembling strategy of AutoGluon.

Secondly, AutoGluon introduces ground-breaking multi-layer stack ensembling. Various basic models make up the first layer, and their outputs are concatenated before being fed into the multiple stacker models that make up the second layer. AutoGluon reuses all its base layer model types as stackers, while conventional stacking techniques utilize simpler models in the stacker than the base layers (with the same hyperparameter settings). The multi-layer stacking approach used by AutoGluon has two features in common with deep learning. The straight transfer of original features to the following layer is comparable to skip connections in residual networks, while layer-wise training is comparable to hidden layers in deep learning. In the final stacking layer, AutoGluon applies ensemble selection to aggregate the stacker models predictions in a weighted manner. Figure 6 shows the multi-layer stacking strategy of autoGluon.

K-fold ensemble bagging, a straightforward ensemble technique that lowers the variance in the generated predictions, helps AutoGluon perform even better. This approach involves randomly dividing the data into k separate units, and then training the model on each k unit using a new set of test data. Each model generates out-of-fold (OOF) predictions based on the validation data, and AutoGluon bags all models.

Overfitting is avoided by repeating the k-fold bagging procedure on n separate random divisions of the training data and averaging all OOF predictions across the bags. Predicting how many bagging rounds can be completed during the allocated training period yields the number of repetitions, n. Averaging over several k-fold bags, OOF forecasts have substantially less variance and are less likely to overfit. According to the authors, the n-repeated k-fold bagging procedure is beneficial for smaller datasets when OOF overfitting occurs since OOF data sizes are constrained. The above innovations make the AutoGluon-trained models faster, more robust, and significantly more accurate.

### 2.6. Model performance

The model performance has been evaluated on training and testing accuracy, Area Under Curve (AUC), sensitivity, and specificity. Five-fold cross-validation was applied to assess the performance of traditional ML models, and for autoML, we rely on the respective inbuilt cross-validation mechanisms. The training accuracy demonstrates the performance of the model on which the model was trained while the testing accuracy is the performance on the external dataset. Area Under the ROC Curve is an important metric in the clinical domain as it provides an aggregate measure of performance across all thresholds (32). The closer the curve approaches the left corner of the graph, the better its discriminating capability. The diagonal reference line on the AUC curve represents a random region where the model is unable to classify favorable or unfavorable outcomes (sensitivity = specificity).

## 3. Results

The total training time for 71 patients using the best-performing model (AutoGluon) was 208.59s and the prediction time for 85 patients was 0.045s.

### 3.1. Comparison of traditional ML and autoML for predicting mRS at discharge

The accuracy of various conventional ML and autoML methods are shown in Table 2. The training accuracy was high among all the conventional ML methods, ranging from 89.49% for SVM to 99.99% for the decision tree classifiers; however, the testing accuracy was suboptimal. The decision tree classifier showed an accuracy of 74.11% while it was only 57.65% for SVM. Other classifiers accuracies lay between these two extreme values. On the contrary, autoML methods displayed good performance in training datasets, with the lowest accuracy of 89.93% for the auto-H2O method to the highest of 94.09% for the AutoGluon method. Other classifiers such as auto-sklearn and TPOT demonstrated an accuracy of 94.03 and 93.69%, respectively. Testing accuracy was highest for AutoGluon which showed an accuracy of 88.23%, followed by MLJAR and auto-sklearn, which had an accuracy of 84.7 and 83.53% respectively. The accuracy for other classifiers was less (76.47 and 72.9% for TPOT and H2O, respectively).

**Table 2:** Performance of various autoMLs and traditional ML algorithms for predicting mRS at discharge.

**Sr. No**	**Models**	**Training accuracy**	**Testing accuracy**	**AUC**	**Sensitivity**	**Specificity**
1	Auto-Gluon	94.09	88.23	0.95	0.74	0.91
2	MLJAR	logloss 0.22^*^	84.7	0.85	0.83	0.89
3	Auto-Sklearn	94.03	83.52	0.87	0.73	0.89
4	TPOT	93.69	76.47	0.91	0.44	0.75
5	Decision tree classifier	99.99	74.11	0.83	0.78	0.89
6	H2O	89.93	72.9	NA*	0.53	0.84
7	Logistic regression	89.05	65.88	0.78	0.53	0.77
8	Random forest	68.7	65.88	0.65	0.34	0.67
9	kNN	92.12	64.7	0.69	0.46	0.74
10	SVM	89.49	57.64	0.73	0.42	0.71

The values of AUC, Sensitivity, and Specificity are for the testing set. *Due to the technical limitation of the concerned autoML, the values could not be calculated.

### 3.2. Comparison of traditional ML and autoML for predicting mRS at 3 months

AutoML methods consistently outperformed traditional ML approaches in the prediction of clinical prognosis at 3 months (Table 3). Similar to earlier results, traditional ML had high training accuracy of 92 to 99%, while a similar optimism was not evident in the testing phase. The highest accuracy was obtained with the SVM classifier (76.5%), while other classifiers demonstrated even worse performances (72.83% for logistic regression and random forest, 69.13% for decision tree classifier, and 51.85% for knn classifier). Accuracy for autoML methods varied from a low score of 72.8% (H2O) to a high score of 85.18% (MLJAR). Auto-gluon was the second-best performing autoML method, with an accuracy of 83.18% while other auto classifiers had an accuracy of 82.7 and 76.54%, respectively.

**Table 3:** Performance of various autoMLs and traditional ML algorithms for predicting mRS at 3 months.

**Sr. No**.	**Models**	**Training accuracy**	**Testing accuracy**	**AUC**	**Sensitivity**	**Specificity**
1	Auto-Gluon	93.12	83.95	0.96	0.75	0.85
2	MLJAR logloss	0.15*	85.18	0.91	0.83	0.91
3	Auto-Sklearn	96.6	82.7	0.89	0.75	0.88
4	TPOT	96	76.54	0.9	0.7	0.85
5	H2O	70.67	72.8	NA*	0.63	0.83
6	Decision tree classifier	99.99	69.13	0.76	0.72	0.85
7	Logistic regression	91.33	72.83	0.89	0.69	0.84
8	Random forest	98.45	72.83	0.89	0.68	0.84
9	kNN	92	51.85	0.67	0.42	0.72
10	SVM	93.11	76.54	0.87	0.72	0.86

The values of AUC, sensitivity, and specificity are for the testing set. ^*^Due to the technical limitation of the concerned autoML, the values could not be calculated.

### 3.3. Area under the curve, sensitivity, and specificity of the model for prediction of mRS at discharge and at 3 months

The AUC for mRS prediction at discharge for the best-performing model (AutoGluon) was 0.95, with sensitivity and specificity of 0.74 and 0.89, respectively. For other autoML methods, the AUC ranged between 80 and 90, while it was generally low for traditional ML techniques. The observation was similar for mRS prediction at 3 months as well, where autoML methods had a high AUC in the range of 0.89–0.96, which was higher than the traditional ML approaches. The sensitivity and specificity of MLJAR and AutoGluon, the two methods that had high predictive accuracy, were 0.83 and 0.9 vs. 0.81 and 0.90, respectively. Tables 2, 3 demonstrate the AUC, sensitivity, and specificity values of all the ML methods evaluated in this study. Supplementary Figures 1, 2 show ROC curves for select autoML and traditional ML methods evaluated in this study.

## 4. Discussion

In several domains, especially medical and clinical, understanding why a model produces a specific prediction can be just as crucial as obtaining accurate predictions. There is a conflict between accuracy and interpretability since the best performance is sometimes attained by complex models, such as ensembling and deep neural networks, that even experts have trouble understanding. The capacity to accurately interpret a prediction model's output fosters the necessary clinical confidence, offers perception into how a model may be improved, and aids comprehension of the process being modeled. In this regard, we explored a popular prediction interpretation framework known as SHAP (SHapley Additive exPlanations) (33), which interprets each feature's importance value, providing insights into the decision-making approach of the model. Most AutoMLs have integrated the SHAP framework into their respective codebase to provide explainability and interpretability.

**Figure 7:**
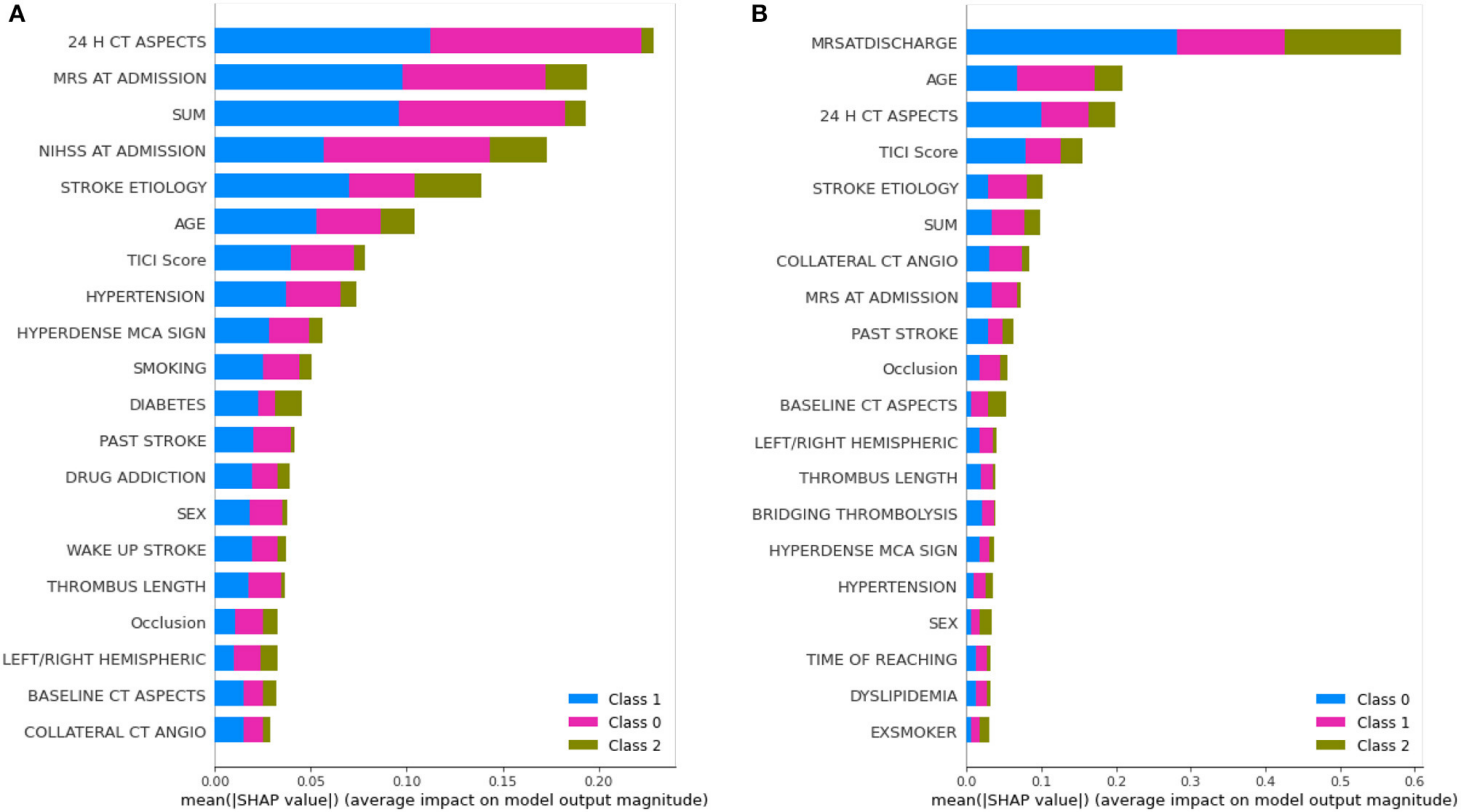
Feature importance of various variables as interpreted by SHAP for mRS at discharge and 3 months by AutoGluon. SUM represents the time taken for recanalization from the onset of stroke. **(A)** mRS at discharge. **(B)** mRS at 3 months.

**Figure 8:**
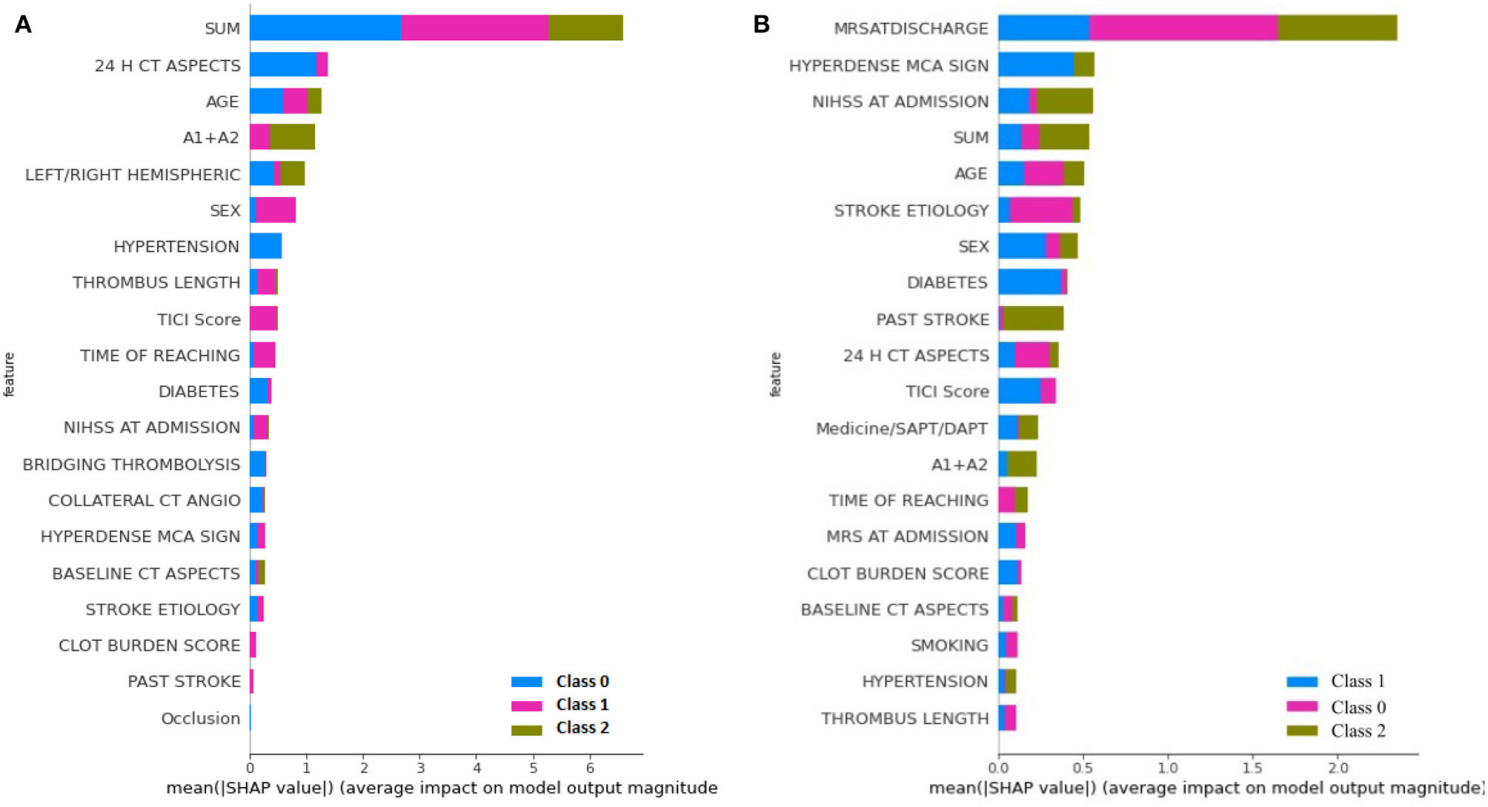
Feature importance of various variables as interpreted by SHAP for mRS at Discharge and 3 months by MLJAR. SUM represents the time taken for recanalization from the onset of stroke. **(A)** mRS at discharge. **(B)** mRS at 3 months.

The learning capacity of a model is dependent on its ability to learn the importance of features in predicting a class. While AutoML constructs complex models by ensembling, it also prioritizes various features as part of feature selection with the aim to improve on the predictions. AutoGluon was the best-performing model on mRS at discharge and MLJAR on mRS at 3 months. Figures 7, 8 show the feature importance of two top-performing models. For the prediction of mRS at discharge by AutoGluon (Figure 7A), the 24-h ASPECTS score had the highest impact. Other features in the order of reducing importance include stroke severity at the onset, time taken for recanalization from the onset of stroke, stroke etiology, age, TICI score, hypertension, hyperdense MCA, and smoking. For the prediction of mRS at 3 months by MLJAR (Figure 8B), mRS at discharge had the highest impact. Other important features in decreasing order were the hyperdense MCA sign, NIHSS at admission, time taken for recanalization from the onset of stroke, age, and stroke etiology.

Comparing the feature importance of the two best-performing models, we may note that for predicting mRS at discharge, 50% of the features in the Top 10 and 80% of the features in the Top 20 are the same. Similarly, for predicting mRS at 3 months, 60% of the features in the Top 10 and 80% of the features in the Top 20 are the same although the relative importance of features in both cases varies. This provides greater insight into the decision-making process of the models and may be a first step toward objectively defining a relative importance score to variables in predicting the modified Rankin Score.

**Figure 9:**
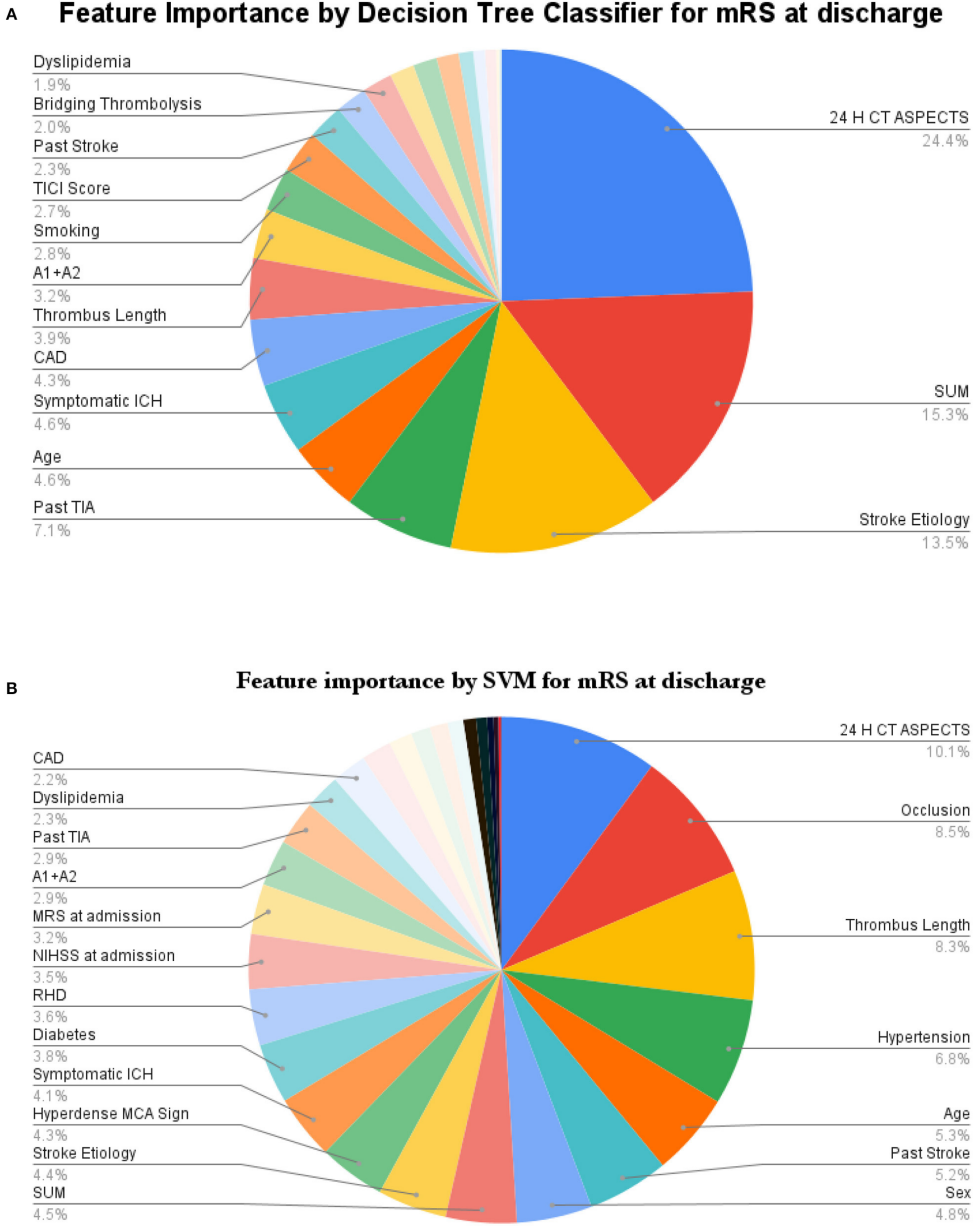
Feature importance as interpreted for mRS at discharge by **(A)** Decision Tree Classifier and **(B)** SVM.

**Figure 10:**
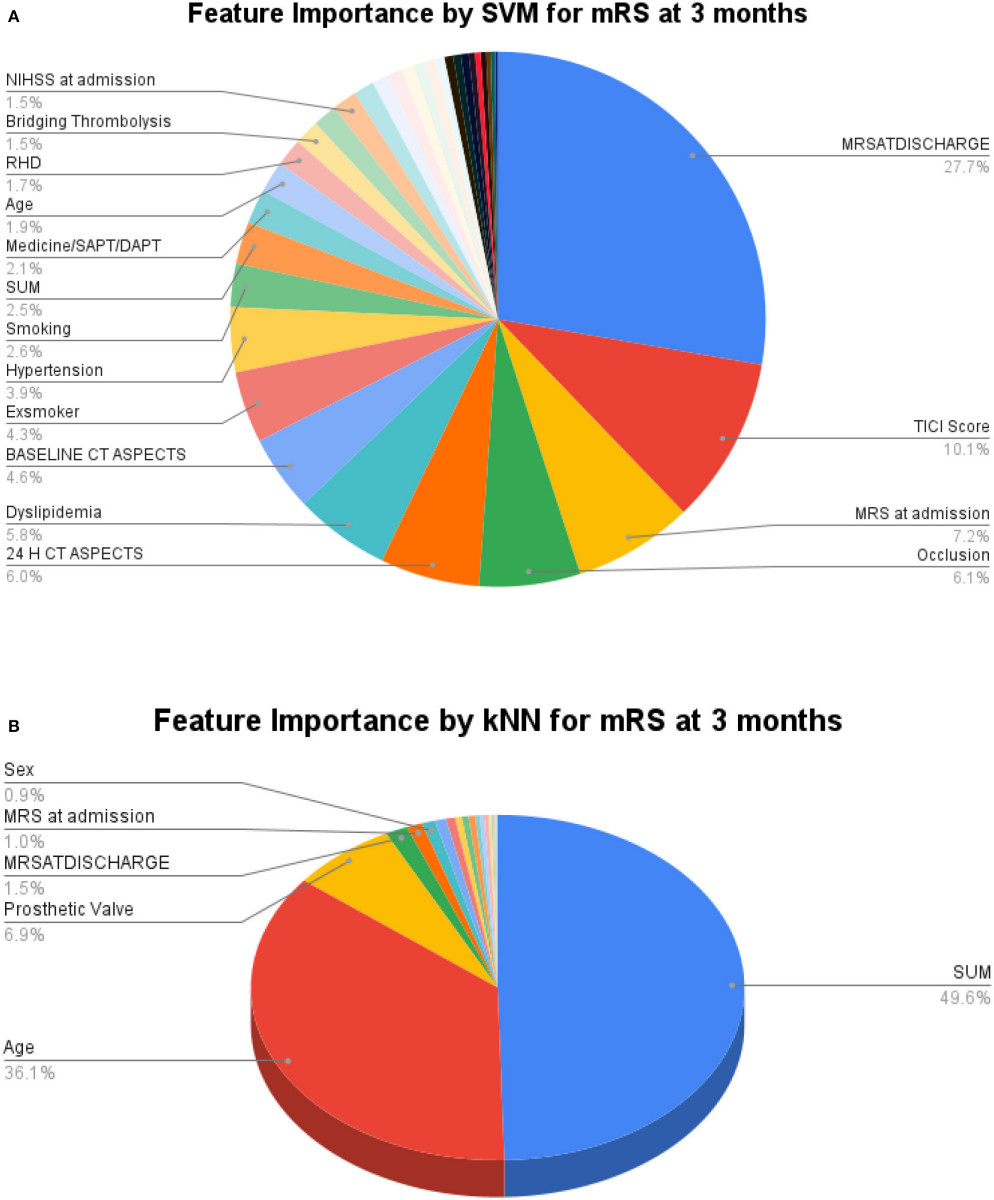
Feature importance as interpreted for mRS at 3 months by **(A)** SVM and **(B)** kNN.

For greater insights into the predictive capability of traditional machine learning algorithms, we investigated the feature importance of these models. Figure 9 shows the feature importance of the decision tree classifier and SVM, which are two of the extreme performing traditional machine learning algorithms for mRS at Discharge. Similarly, Figure 10 shows the feature importance of SVM and kNN, which are two of the extreme performing traditional machine learning algorithms for mRS at 3 months. It was observed that the order of relative importance and number of variables considered in model decision-making play a crucial role in model prediction. For example, comparing Figures 7A, 9A, one may observe that while AutoGluon and the decision tree classifier have almost the same number of features (20 in this case), their relative importance is a decisive factor in the model prediction. While autoGluon has attributed 24 H CT ASPECTS the highest importance, the decision tree classifier has attributed it to time for recanalization from the onset of stroke (SUM). Forty percent of the features in the Top 10 and 60% of the features in the Top 20 are matching between the best-performing ML model and the best-performing AutoML model. These matches are less than the one compared between the best-performing AutoML models. The poor performance of kNN in predicting mRS at 3 months may be attributed to the incorrect feature importance assigned to various clinical parameters for the prediction of mRS scores (Figure 10B). While the best-performing autoML methods have their feature importance aligned to the clinical methodology of predicting mRS at discharge and 3 months, the under-performing machine learning algorithms have failed to develop a decision model as per clinical requirements. The interpretation of the model's output could go a long way in fostering confidence among clinicians and enable greater adaptation of autoML in clinical practice.

From the clinical perspective, a clinician also uses the same factors to select and counsel patients prior to the procedure although much more subjectively than that possible with the use of autoML algorithms. A pre-hand knowledge of which factors and the degree of the effect of the functional outcome of mechanical thrombectomy procedure will help in providing a customized approach to each patient. The application of the model into routine clinical use would ensure that those patients who would gain maximum benefit out of the procedure are offered the treatment timely. It will also help to avoid unnecessary invasive interventions in those who are unlikely to improve post-thrombectomy procedures. Additionally, it will aid the clinicians in better counseling of stroke patients and their families regarding the prognosis.

Several striking features distinguish this study from the previous related works. In the most recent study, (34) manually developed an ensemble of seven ML models with nine clinical features. In contrast, we explored automated ensembling, without human intervention with an exhaustive list of 34 treatment variables. The other advantage of autoML is its ability to handle missing values either by imputing or categorizing them as “Unknown” to handle real-world scenarios. Furthermore, training the models on a small training set and testing it on an equal number of samples, as in the real-world scenario, helps to test the generalizability capacity of the models. In this respect, the prediction capability of autoML vis-a-vis traditional ML algorithms can be appreciated. The integration of SHAP to autoML frameworks has filled the gap between accuracy and interpretability giving clinicians the necessary confidence in using the developed models as decision support systems. Automated feature selection and model ensembling would go a long way in decreasing human intervention and manual effort, thus promoting the adoption of autoML across domains.

There are a few limitations to our study. Firstly, since it was a single-center retrospective study, the testing sample although equivalent to the training sample, can further be enhanced and tested for real-world deployments. Secondly, as evident from Figure 7, the autoML frameworks considered only 20 features of the given 34 features in their decision-making. Prediction capability through the remaining 14 features may be studied in future works.

## 5. Conclusion

In conclusion, our study demonstrates the robustness, efficiency, and potential of autoML over traditional ML algorithms. Even with an exhaustive list of treatment variables and a small training sample size, autoML outperformed traditional ML models in predicting functional outcomes. The promising results suggest that further development and deployment of autoML frameworks in the clinical domain could assist clinicians in early prognosis, customized treatments, and improved counseling for patients and their families.

## Data availability statement

The raw data supporting the conclusions of this article will be made available by the authors, without undue reservation.

## Ethics statement

The studies involving humans were approved by Institute Ethics Committee, Sree Chitra Tirunal Institute of Medical Sciences and Technology, Trivandrum, Kerala, India. The studies were conducted in accordance with the local legislation and institutional requirements. Written informed consent for participation was not required from the participants or the participants' legal guardians/next of kin in accordance with the national legislation and institutional requirements. Written informed consent was not obtained from the individual(s) for the publication of any potentially identifiable images or data included in this article because as it was a retrospective study, the need for informed consent was waived off by the Institute Ethics Committee.

## Author contributions

RR: Data curation, Formal analysis, Software, Visualization, Writing—original draft, Writing—review and editing. SK: Conceptualization, Data curation, Formal analysis, Funding acquisition, Investigation, Supervision, Writing—original draft, Writing—review and editing. JM: Investigation, Project administration, Resources, Validation, Visualization, Writing—review and editing. PS: Data curation, Supervision, Validation, Writing—review and editing.
