# Supplementary material for: AutoML accurately predicts endovascular mechanical thrombectomy in acute large vessel ischemic stroke
Source: Front Neurol. 2023 Sep 28;14:1259958. doi: 10.3389/fneur.2023.1259958 (PMC10569475; doi:10.3389/fneur.2023.1259958)
Supplement: Supplementary file 1 [file Data_Sheet_1.PDF]

Supplementary Material

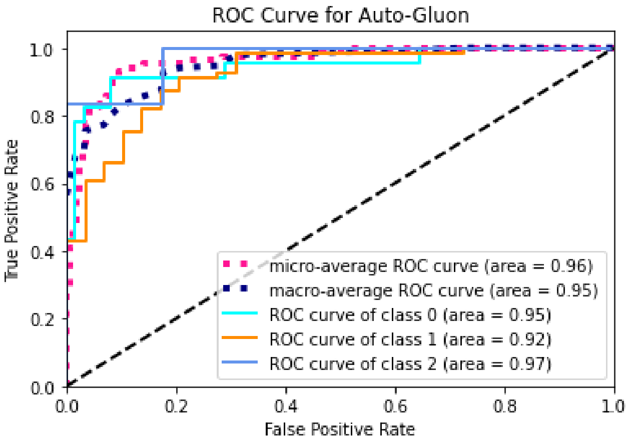

Figure 1a. AutoGluon

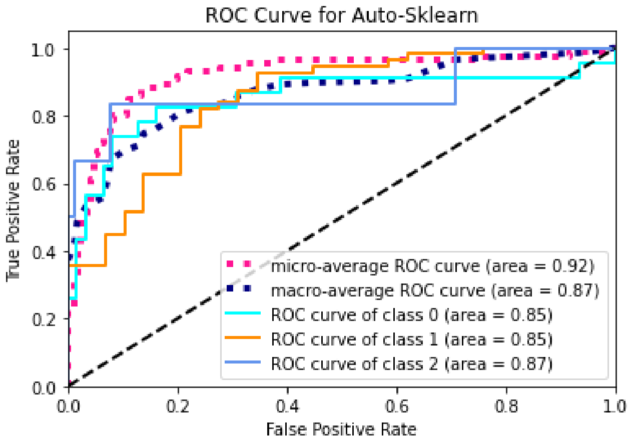

Figure 1b. Auto-sklearn

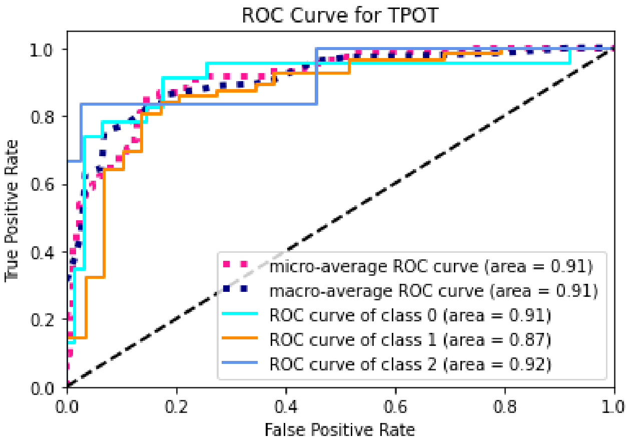

Figure 1c. TPOT

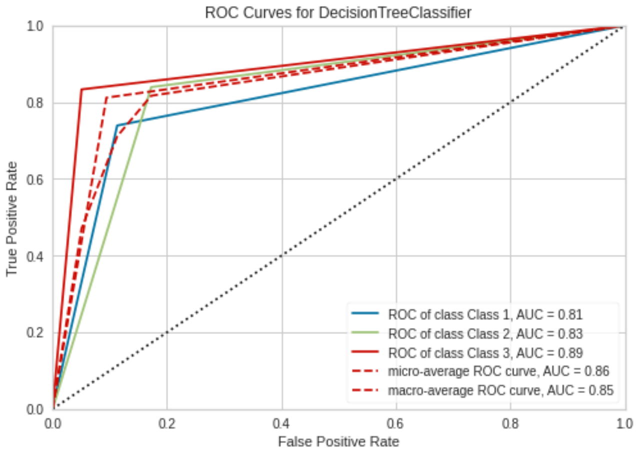

Figure 1d. Decision Tree Classifier

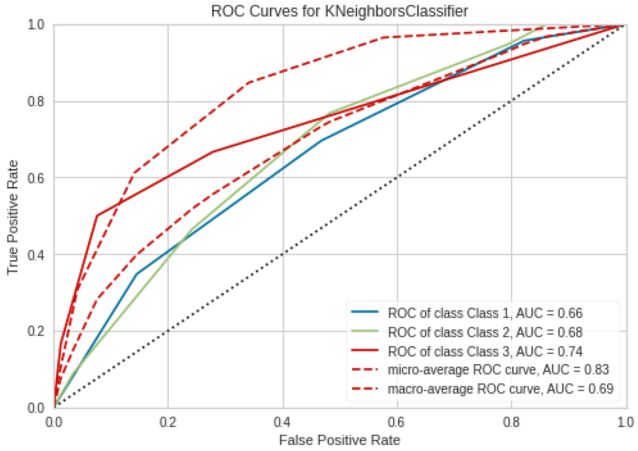

Figure 1e. k Nearest Neighbours

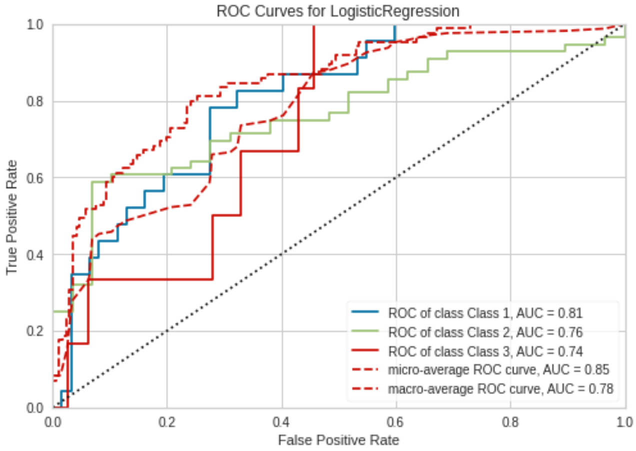

Figure 1f. Logistic Regression

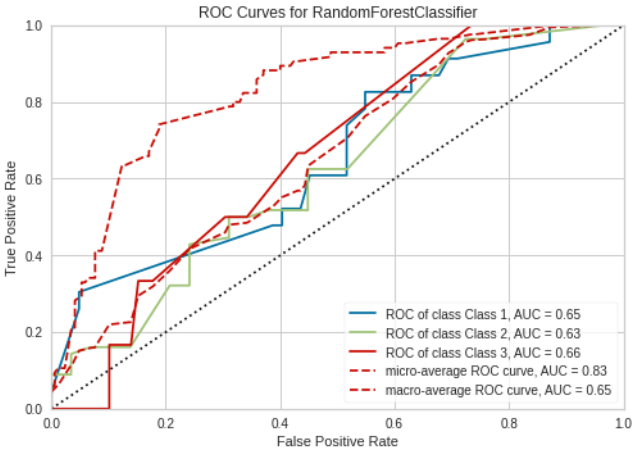

Figure 1g. Random Forest

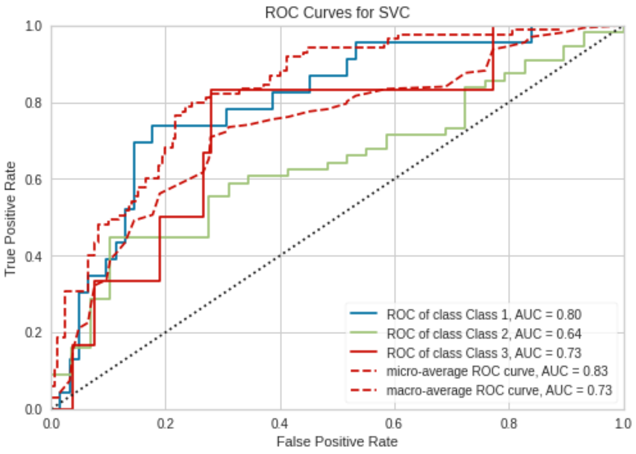

Figure 1h. Support Vector Machine

Figure 1. ROC curves for select autoML and traditional ML models for prediction of mRS at discharge

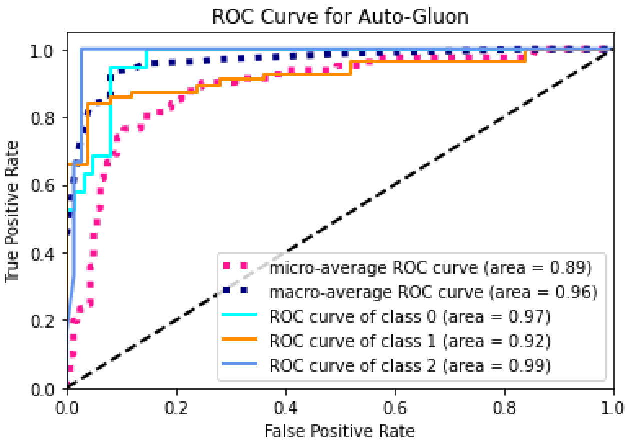

Figure 2a. AutoGluon

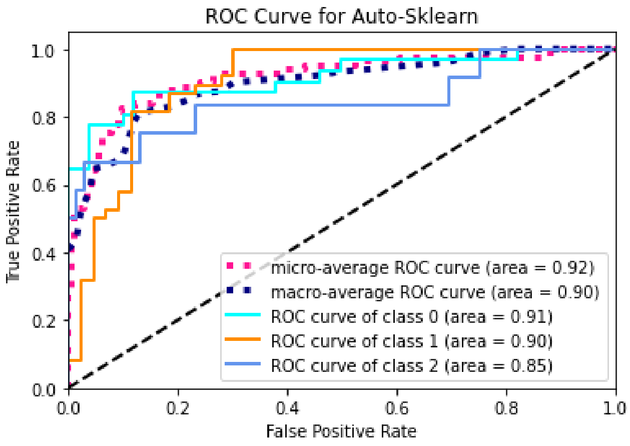

Figure 2b. Auto-sklearn

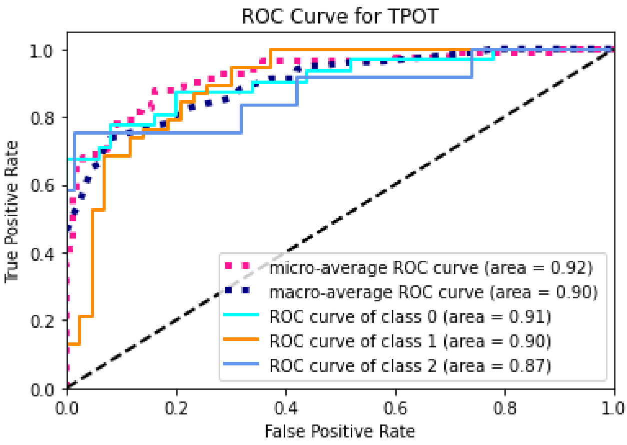

Figure 2c. TPOT

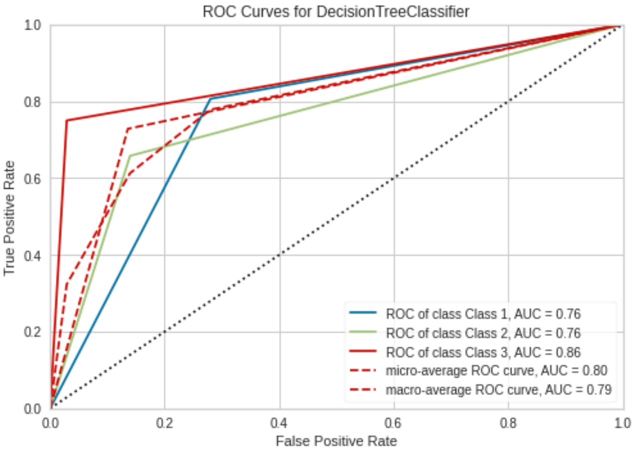

Figure 2d. Decision Tree Classifier

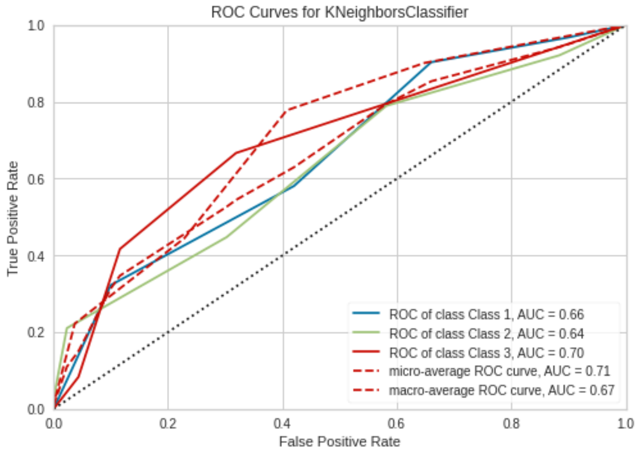

Figure 2e. k Nearest Neighbours

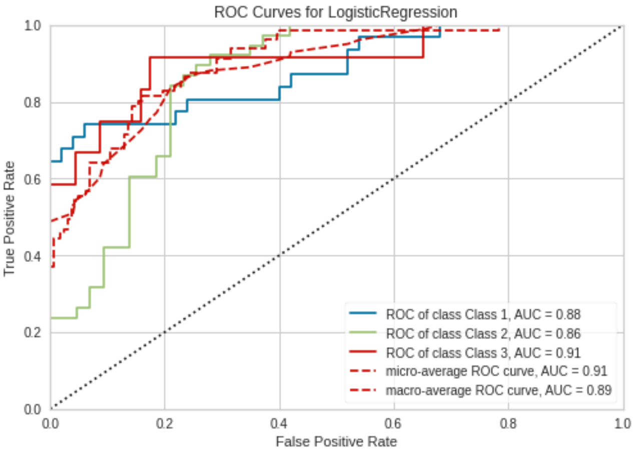

Figure 2f. Logistic Regression

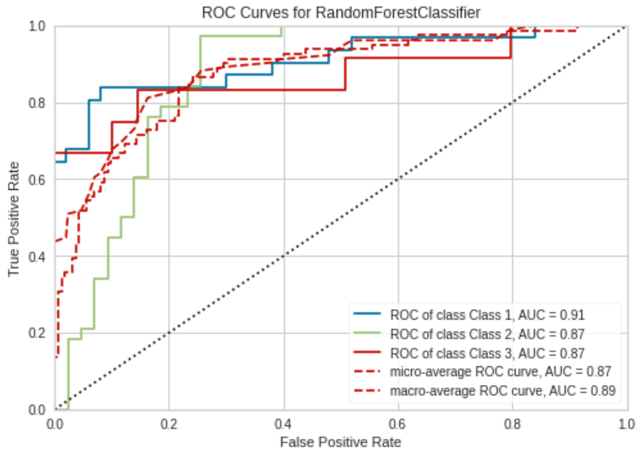

Figure 2g. Random Forest

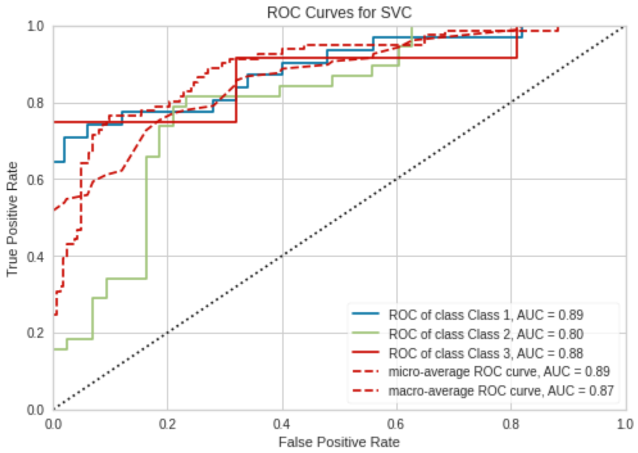

Figure 2h. Support Vector Machine

Figure 2. ROC curves for select autoML and traditional ML models for prediction of mRS at 3 months

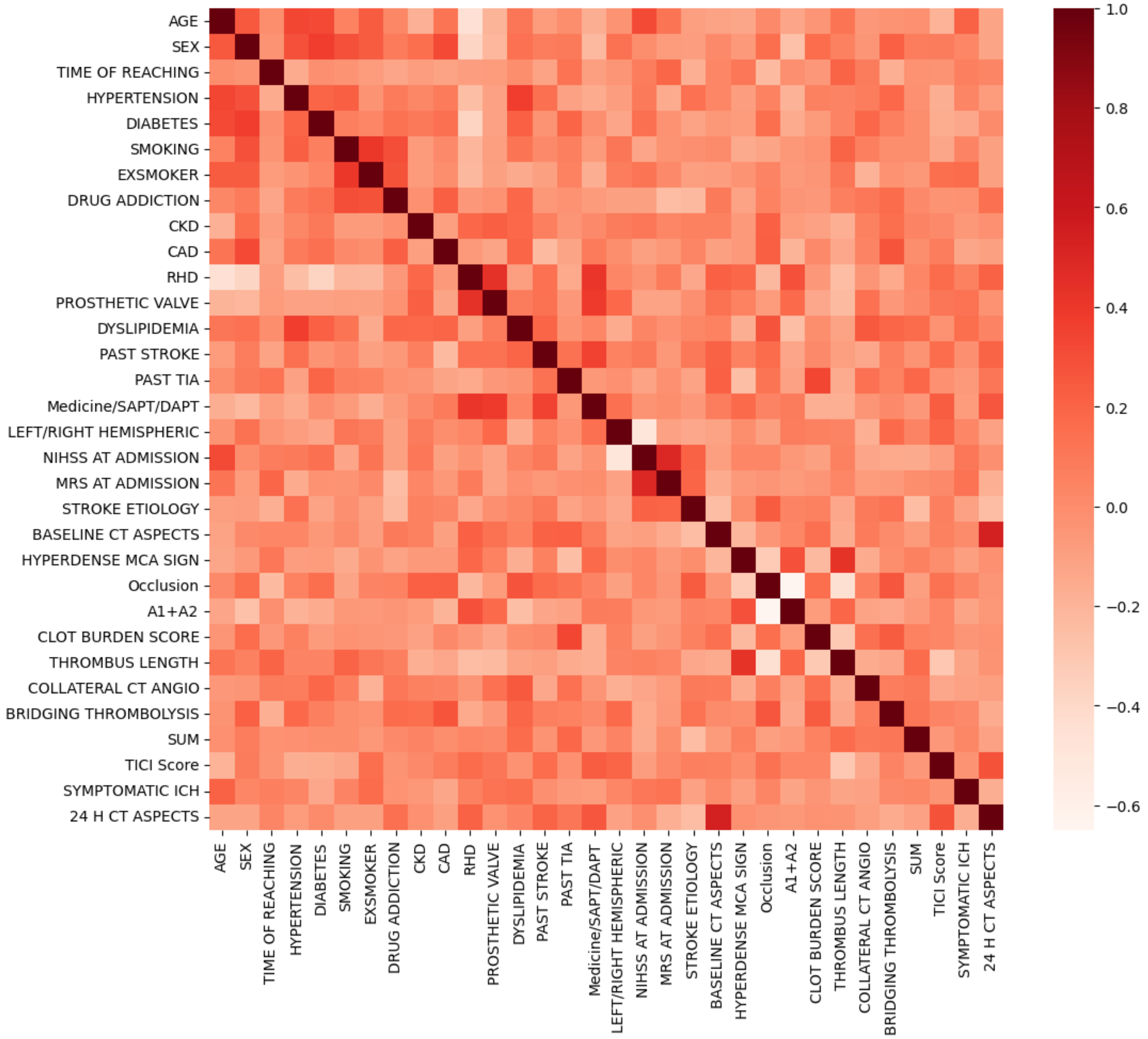

Figure S3. Correlations between various treatment variables.

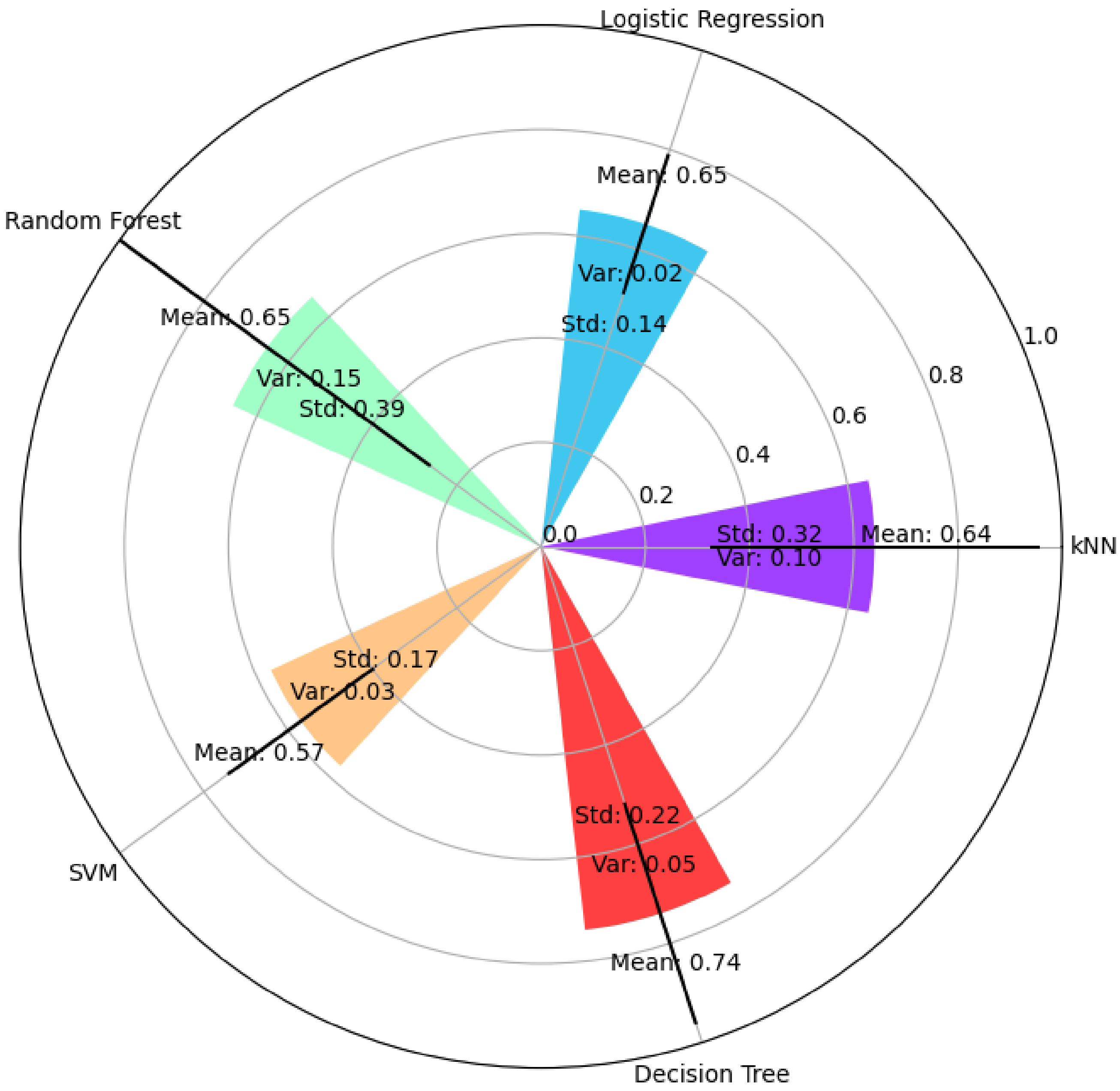

Figure S4. Five-fold cross-validation results for traditional ML algorithms for mRS at Discharge

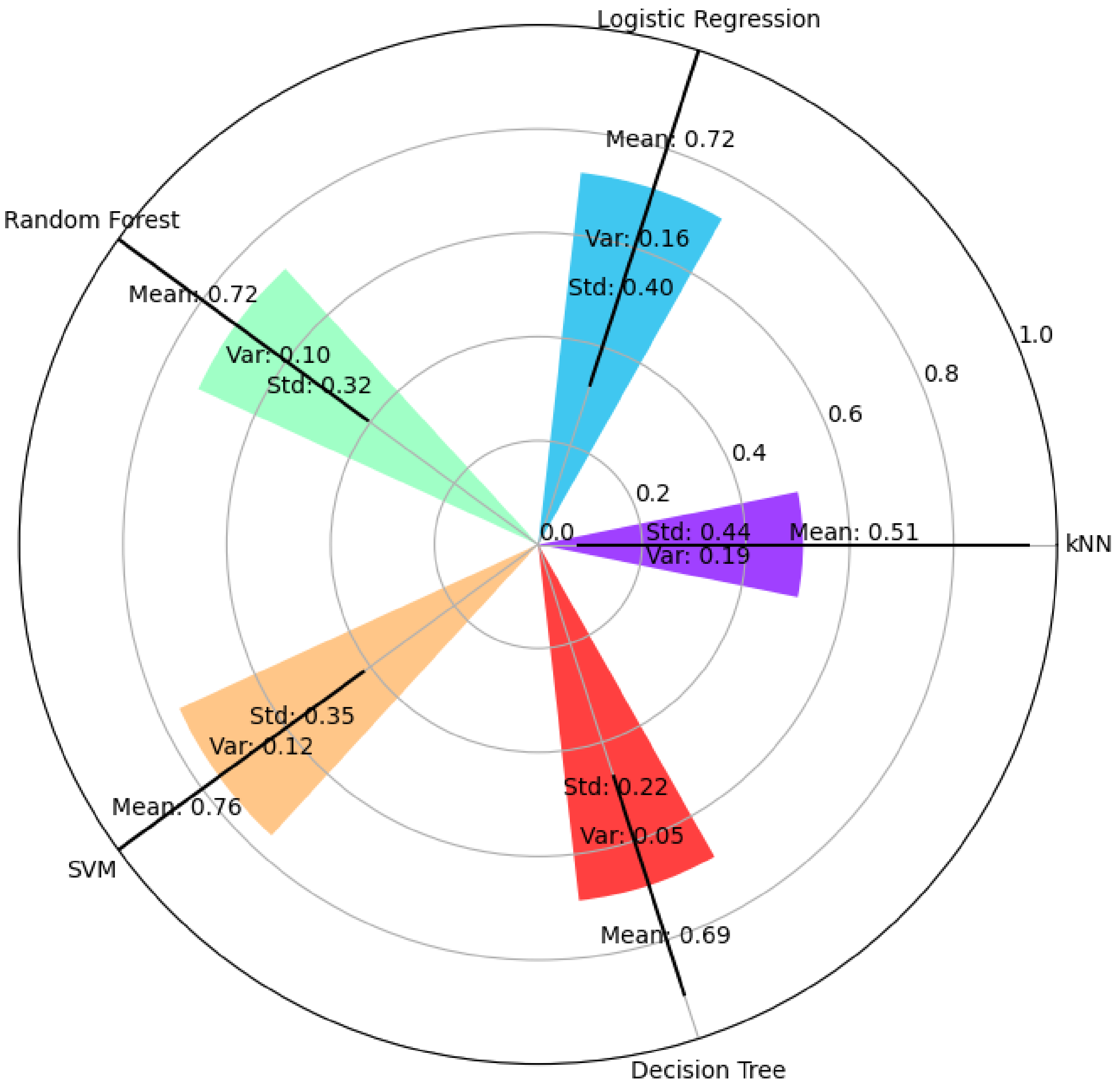

Figure S5. Five-fold cross-validation results for traditional ML algorithms for mRS at 3 months
